# Moderate alcohol drinking is not associated with risk of depression in older adults

**DOI:** 10.1038/s41598-018-29985-4

**Published:** 2018-07-31

**Authors:** Esther García-Esquinas, Rosario Ortolá, Iñaki Galán, Hosanna Soler-Vila, Martín Laclaustra, Fernando Rodríguez-Artalejo

**Affiliations:** 1 Department of Preventive Medicine and Public Health, Universidad Autónoma de Madrid and Idipaz, Madrid, Spain; 2 CIBER of Epidemiology and Public Health (CIBERESP), Madrid, Spain; 3 National Center for Epidemiology. Instituto de Salud Carlos III, Madrid, Spain; 4 Aragon Institute for Health Research (IIS Aragón), Translational Research Unit, Hospital Universitario Miguel Servet, Zaragoza, Spain; 5 CIBER of Cardiovascular Diseases (CIBERCV), Madrid, Spain

## Abstract

The scarce research on the effects of moderate alcohol consumption on mental health among older adults suggests a protective effect against depression. We prospectively examined the association between patterns of moderate alcohol consumption, depression and psychological distress, using information from 5,299 community-dwelling older adults from the ELSA and Seniors-ENRICA cohorts. A Mediterranean drinking pattern (MDP) was defined as moderate alcohol intake (<40 g/day for men; <24 g/day for women) with a preference for wine and drinking only with meals. Depression was ascertained with the 10-item Geriatric Depression Scale (GDS-10), a self-report of clinically-diagnosed depression, or being on anti-depressant medication (Seniors-ENRICA); and with the 8-item Center for Epidemiologic Studies Depression Scale (CES-D) (ELSA). Psychological distress was assessed with the General Health Questionnaire-12 (GHQ-12). Compared to never drinkers, moderate drinkers showed comparable scores on the ENRICA-GDS-10 (PRR (95%CI): 1.03 (0.84–1.26)), the ENRICA-GHQ-12 (0.88 (0.73–1.06)), the ELSA-CES-D (0.92 (0.79–1.06)) and the ELSA-GHQ-12 (0.75 (0.55–1.01). The MDP was not associated with the GDS-10 or GHQ-12 scores, or with clinically-diagnosed depression; however drinkers with a preference for wine showed an increased number of psychological distress symptoms (1.31 (1.03–1.66)). In conclusion, we found no consistent protective association between moderate alcohol consumption and depression in older adults.

## Introduction

Among older adults, depression is the most common mental illness^1^. According to the Global Burden of Disease Study, in 2015 the number of Disability Adjusted Life Years (DALYs) for depressive disorders in older adults ranged from 750 per 100,000 among those aged 80 and over to 1050 per 100,000 among those aged 60–65^2^. Depression in these age groups frequently coexists with other chronic diseases, cognitive impairment, and psychosocial problems (i.e., isolation or economic impoverishment). Moreover, depression in older adults is associated with a higher use of healthcare services^3^, and is a risk factor of physical function limitations^4^, suicide^5^, and premature mortality^6,7^.

Heavy alcohol consumption significantly contributes to the burden of disease in many countries^8^. Major illnesses associated with alcohol intake include cancers (e.g., malignancies in the oral cavity, pharynx, larynx, esophagus, liver, colon, rectum, female breast)^9^, cardiovascular diseases (e.g., hypertension, hemorrhagic stroke, atrial fibrillation)^8^, and gastrointestinal conditions (e.g., alcoholic hepatitis, cirrhosis, pancreatitis)^10^. Nevertheless, moderate alcohol consumption has been associated with a reduction of some health risks; for instance, compared to non- drinkers, moderate alcohol drinkers display a reduced risk of coronary artery disease^11^, ischemic stroke^11^, heart failure^11^, and type 2 diabetes^12^.

A large body of research has traditionally documented the deleterious relationship between alcohol abuse and depression^13^. However, studies have seldom examined the psychological and mental health effects of light to moderate alcohol consumption, and the scarce longitudinal reports have rendered inconsistent results^14–19^. Thus, the main objective of this work was to examine the prospective association between moderate alcohol intake and depression or psychological distress in two cohorts of community-dwelling older adults: the Seniors-ENRICA (Study on Nutrition and Cardiovascular Risk in Spain) and the ELSA (English Longitudinal Study of Ageing). Given that the effects of alcohol on mental health might be context-specific, analyzing data from these two cohorts may facilitate determining whether any associations found differ between “Mediterranean” countries like Spain, where alcohol is traditionally used as a nutritional element and most drinking occurs during meals, and “Non-Mediterranean” countries like England, where alcohol is consumed in greater amounts and mainly used as a psychotropic-relaxant. In this work we also assessed the impact of the Mediterranean Drinking Pattern (MDP) on depression and psychological distress. The MDP, characterized by moderate alcohol intake, a preference for wine, and drinking only with meals, is the traditional pattern of alcohol consumption in Spain and other regions of the Mediterranean basin^20^. Previous studies among older adults have reported the MDP to be associated with a reduced risk of functional limitations^21^, frailty^22^ and falls^23^, and with better scores on the Physical Component Summary of the12-item Short Form Health Survey^24^. However, its effects over mental health are unknown.

## Methods

### Study population and design

#### Seniors-ENRICA cohort

This cohort was established during 2008–2010 with individuals selected by multi-stage stratified random sampling from the non-institutionalized Spanish population aged ≥60 years (wave 1)^25^. For these analyses, we used information from 2,519 subjects participating in wave 2 (2012) and followed through wave 3 (2015)^26^. In these two waves, information regarding sociodemographic variables, health behaviors and morbidity was collected using a computer assisted telephone interview. There is evidence from Spanish population studies that telephone surveys yield similar distributions of health behaviors and morbidities as face-to-face interviews^27,28^. In particular, the Spanish telephone-administered geriatric depression scale has shown a high correlation with the validated personal administration of the scale^27^. An advantage of the telephone-administered geriatric depression scale is that it may increase participation, as depressed individuals are less likely to respond to fac-e-to-face questions on depression^27^. After telephone interviews, a diet history and a physical exam were performed by trained staff during at-home visits.

From the initial sample of 2,519 participants in 2012, 82 (3.3%) died and 616 (24.5%) were lost to follow-up; thus, in 2015 data on depression and psychological distress came from 1821 individuals. Participants remaining in the study were slightly younger, had higher educational level, fewer comorbidities and lower scores in the GDS and GHQ-12 scales. Never drinkers and ex-drinkers were more likely to be lost to follow-up. All participants gave informed written consent, and the Clinical Research Ethics Committee of the *La Paz* University Hospital in Madrid approved the study. All methods were performed in accordance with the relevant guidelines and regulations.

#### ELSA cohort

This cohort comprises people aged ≥50 years living in private households in England^29^. The present research analyzed data from Waves 0, 1 and 3. As in previous reports^30,31^, a combination of Wave 0 (drawn from households that had previously responded to the Health Survey from England in 1998, 1999 or 2001) and Wave 1 (which included those households from Wave 0 where at least one member was aged ≥50 years between March 2002 and March 2003), was used to define the baseline sample (n = 11,391). Information from the initial sample was collected using a personal face-to-face Computer Assisted Personal Interview, a nurse visit and a self-completion questionnaire. From the initial sample, we excluded those individuals aged <60 years (n = 4,166).

During follow-up, 241 participants died (3.3%), 1928 (26.7%) refused to participate or could not be contacted, and 488 (6.8%) became ineligible (mainly because they had moved outside of Britain or had been institutionalized). Thus, in 2006–2007, information on depression and physiologic distress was collected from 4,568 participants aged ≥60 years. Participants remaining in the study were more frequently women, younger, had higher educational level, lower tobacco consumption, fewer comorbidities and lower scores in the CES-D. The National Research Ethics Service (MREC/01/2/91) provided the ethical approval for ELSA.

### Study variables

#### Alcohol consumption

In the Seniors-ENRICA study, alcohol consumption was estimated based on a validated diet history, developed from the one used in the EPIC cohort study in Spain^32^. The diet history collected consumption data for 34 alcoholic beverages and used photographs depicting different glass sizes to help quantify serving sizes. Alcohol content, in grams, was then estimated using standard composition tables. According to the average alcohol intake, participants were classified as (1) never drinkers (this category also included occasional drinkers with average intake close to zero), (2) ex-drinkers (those reporting having stopped drinking for at least a year before the interview), (3) moderate drinkers and 4) heavy drinkers. We set the threshold between moderate and heavy drinking at ≥40 g/day for men and ≥24 g/day for women^33^. Among drinkers (categories 3 and 4 in the previous classification), a preference for a specific type of alcoholic beverage (wine or other) was acknowledged when more than 80% of alcohol intake was derived from such drink^22^. According to the consumption of alcohol with meals (lunch or dinner), drinkers (categories 3 and 4) were also classified into three groups: those who drank exclusively during mealtimes, those who drank exclusively outside meal times, and those who drank at any time. Finally, a Mediterranean Drinking Pattern (MDP) was defined as moderate average intake of alcohol (and no binge drinking), with wine preference and alcohol consumption only with meals^22^.

For the ELSA-cohort analyses, information on alcohol consumption and frequency was derived from Wave 0 but not from Wave 1 because this wave collected information only on the frequency of drinking. According to the average alcohol intake, and having defined 10 grams of pure alcohol as equivalent to an alcohol unit, participants were classified as never drinkers, ex-drinkers, moderate drinkers, and heavy drinkers. The threshold between moderate and heavy drinking was set at 28 units of alcohol/week in men and 21 units/week in women.

#### Depression

Depression was ascertained with three instruments:

- Geriatric Depression Scale (GDS): To assess depressive symptoms, the Seniors-ENRICA study used the 10-item version of the GDS^34^. Individuals missing ≥2 items were excluded from the analyses. A higher score in the GDS indicates a greater level of depression.
- Center for Epidemiologic Studies Depression Scale (CES-D): The ELSA study used the 8-item version of the CES-D)^35^. A higher score in the CES-D indicates a greater level of depression.
- Self-reported depression: In the Seniors-ENRICA study this variable was defined as a positive answer to the question “Has a doctor ever told you that you have depression” or as being on antidepressant medication.

#### Psychological distress

The Seniors-ENRICA and the ELSA studies used the 12-item version of the General Health Questionnaire (GHQ-12), a screening tool for psychological distress. Individuals missing ≥2 items were excluded from the analyses. Higher scores in the GHQ-12 indicate higher psychological distress.

#### Other variables

Baseline self-reported data on age, sex, educational status, and tobacco consumption were collected in both cohorts. In the Seniors-ENRICA cohort, information on physical activity (metabolic equivalent-hour/week) and on the number of hours/day spent watching television was assessed using validated questionnaires^36,37^. In the ELSA study, participants were asked about the frequency (more than once a week, once a week, one to three times a month, or hardly ever/never) of participation in vigorous-, moderate-, and light-intensity activities. An overall measure of physical activity was generated by multiplying activity frequency by the metabolic equivalent value for each type of activity (6 for vigorous-, 3 for moderate-, and 1.5 for light-intensity activities, respectively). In the Seniors-ENRICA, adherence to the Mediterranean diet was captured using the Mediterranean Adherence Screener (MEDAS) index (excluding the alcohol component)^38^, and energy intake was calculated based on Spanish food composition tables. In both cohorts, baseline weight and height were measured with standard methods. BMI was calculated as (weight in kg)/(height in m)^2^. Normal weight was defined as a BMI <25, overweight as a BMI 25–29.9, and obesity as a BMI ≥30. Finally, in both cohorts, participants also reported whether they had previously been diagnosed with any of the following diseases: cardiovascular disease (ischemic heart disease, stroke, heart failure), diabetes, chronic lung disease (asthma, chronic bronchitis), and osteomuscular disease (osteoarthritis or arthritis); as well as the number of medications they were taking. In the Seniors-ENRICA, study staff compared self-reported information on medication use with medication packages stored at home.

### Statistical analysis

From the initial 1,821 participants in the Seniors-ENRICA cohort, we excluded 200 (11%) subjects with incomplete data on alcohol consumption or potential confounders. Additionally, for analyses on depression we excluded subjects lacking information on the GDS at baseline or follow-up (n = 411; 22.6%). For analyses on psychological distress we excluded individuals with missing data on the GHQ-12 at baseline or follow-up (n = 31; 1.7%). Similarly, from the initial 4,568 participants in the ELSA cohort, 440 (9.6%) subjects were excluded due to incomplete data on alcohol consumption or potential confounders. Also, we excluded individuals with missing information on the CES-D at baseline or follow-up (n = 157; 3.4%) for analyses on depression, and individuals with no data on the GHQ-12 at baseline or follow-up (n = 768; 16.8%) for analyses on psychological distress. Baseline socio-demographic, lifestyle, and clinical characteristics of individuals with and without missing information were similar, although the latter were on average younger, were more likely to be moderate drinkers, and showed a lower prevalence of diabetes and osteomuscular disease.

**Figure 1:**
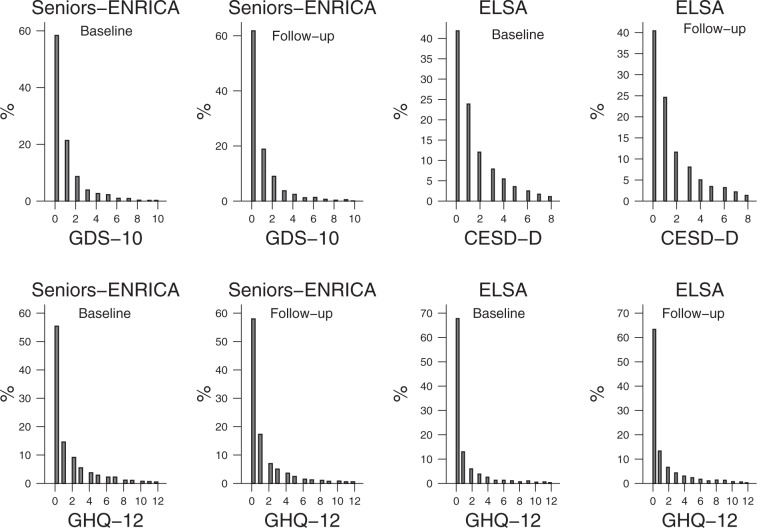
Distribution of depression and psychological distress scales in the Seniors-ENRICA and ELSA cohorts, at baseline and at follow-up.

Associations between alcohol consumption and depression or psychological distress were analyzed with generalized regression models because of the highly skewed distribution of the dependent variables (GDS-10, GHQ-12, and CES-D), which mirrored that of count variables (see Fig. 1). To account for over-dispersion and a higher number of zero values than would be expected under the Poisson distribution, we considered using either negative binomial or zero-inflated negative binomial models. Both approaches were compared using Vuong tests which suggested that zero-inflated negative binomial models provided a better fit of the data. Results were expressed as prevalence rate ratios (PRR) and their 95% confidence interval (CI). PRRs in this context represent the percent change in depression and psychological distress scales for one category of alcohol relative to the reference category, holding other variables constant. When the dependent variable was self-reported diagnosed depression, the analyses were performed using Poisson regression with a robust error variance, and results were expressed as Incidence rate ratios (IRR) and their 95% confidence interval.

We built three regression models with progressive levels of adjustment. Model 1 adjusted for age, sex, education, and the corresponding baseline values of the dependent variables. Model 2 further adjusted for tobacco consumption, physical activity, BMI, cardiovascular disease, diabetes, chronic lung disease, osteomuscular disease, and cancer in both cohorts. Additionally, the Seniors-ENRICA model 2 adjusted for time watching TV, the MEDAS score, total energy intake, and use of sleeping pills or other central nervous system-acting medications. Analyses of the influence of average alcohol consumption and the MDP were conducted in the total sample, whereas analyses of the influence of beverage preference and drinking with meals were performed only among drinkers. Models for specific drinking habits (i.e. beverage preference, drinking with meals or the MDP) were further adjusted for total alcohol consumption (grams/day).

We conducted separate sensitivity analyses. First, we excluded subjects with baseline cardiovascular disease or cancer as well as participants under anxiolytic, antipsychotic, or antiepileptic medication at baseline. Second, to facilitate comparisons between cohorts, we reran analyses with abstainers (never drinkers and ex-drinkers) as the reference category, and reduced the threshold between moderate and heavy intake to 15 g/day for both genders. Third, we split moderate consumption into two categories: below or above the median average alcohol intake. Fourth, we evaluated the risk of depression as defined by a score above 3 in the CESD or GDS using logistic regression. Finally, we modeled the association between alcohol consumption patterns and changes (defined as difference between follow-up and baseline values) in the GDS-10, GHQ-12, and CES-D, using the VCE (bootstrap) command in Stata to estimate confidence intervals based on 100 resamples (see Supplementary Tables 1 and 2). Our results are presented for both sexes combined because sex-alcohol drinking patterns interactions terms included in regression models failed to reach statistical significance and findings were similar in each sex.

Statistical analyses were performed using Stata®, version 13.1.

### Data availability

Data from the Seniors-ENRICA can be accessed upon request to the authors. Data from the ELSA study are available upon request to the UK Data Service

## Results

The prevalence of ex-drinkers, moderate drinkers and heavy drinkers at baseline was 7%, 62% and 5% in Seniors-ENRICA, and 5%, 80% and 10% in ELSA, respectively. and Compared to never-drinkers, ex-drinkers were more often male, had higher education, and were more likely to report unhealthy characteristics (obesity, cardiovascular disease, cancer, and use of sleeping pills and other central nervous system-acting medications) (please see Table 1). Both moderate and heavy drinkers were younger, more frequently male, more educated, and, despite lower levels of physical activity, healthier (lower prevalence of obesity, chronic disease, and medication use, and more favorable baseline scores on the GDS-10, GHQ-12 and CESD) (Table 1). According to the GDS (GDS >3) and CESD (CESD >3) scores, at baseline 7.7% (Seniors-ENRICA) and 14.3% (ELSA) of participants had a diagnosis of depression. Similarly, a physician diagnosis of depression was reported at baseline by 7.5% of participants in the Seniors-ENRICA.

**Table 1:** Characteristics of the study participants by average alcohol consumption and study cohort.

	Seniors-ENRICA				ELSA			
	Never drinker	Ex-drinker	Moderate drinker^a^	Heavy drinker	Never drinker	Ex-drinker	Moderate drinker^a^	Heavy drinker
Female, %	79.1	54.3	39.3	35.2	81.3	59.6	57.5	29.9
Age, years, mean (SD)	71.9 (5.9)	72.5 (6.3)	70.8 (5.8)	70.6 (5.4)	69.0 (7.2)	68.0 (7.2)	67.0 (7.0)	65.9 (7.0)
Educational level, %								
≤Primary	62.9	54.3	45.2	31.0	44.8	39.4	32.7	24.6
Secondary	22.5	24.4	27.6	33.8	37.4	45.2	45.8	46.4
University	14.6	21.3	27.2	35.2	17.8	15.4	21.5	29.0
Current smokers, %	4.7	10.6	11.4	19.7	14.6	22.9	15.1	20.6
Physical activity, MET-h/wk, mean (SD)	64.3 (32.0)	60.3 (31.8)	54.9 (29.5)	49.1 (28.1)				
Physical activity, % lowest quartile					16.9	17.6	25.7	30.4
TV viewing, h/wk	19.4 (10.5)	20.7 (11.9)	19.0 (10.2)	17.2 (8.1)	—	—	—	—
MEDAS score, mean (SD)^b^	4.1 (1.7)	4.2 (1.4)	4.0 (1.6)	3.5 (1.4)	—	—	—	—
Energy intake, kcal/d, mean (SD)	1873 (401)	1948 (485)	2076 (434)	2614 (1403)	—	—	—	—
BMI ≥30 kg/m^2^, %^c^	30.6	35.1	30.9	29.6	29.7	28.2	25.2	22.3
Comorbities, %								
Cardiovascular disease^d^	7.1	9.6	5.1	4.2	12.8	23.4	13.4	11.0
Respiratory disease	11.2	10.6	7.7	8.5	15.5	17.6	14.2	14.6
Osteomuscular disease	69.5	66.0	55.0	40.9	37.9	41.5	34.4	32.8
Diabetes	16.7	23.4	15.7	15.5	8.2	6.9	4.8	4.3
Cancer	3.7	4.3	2.1	0.0	4.6	6.9	6.5	4.6
Sleeping pills, %	21.4	29.8	18.6	15.5				
Other central nervous system-acting drugs,%	37.6	42.6	26.7	22.5	—	—	—	—
Baseline GDS^e^ scores, mean (SD)	1.2 (1.8)	1.5 (2.0)	0.7 (1.4)	0.5 (1.1)	—	—	—	—
Baseline GHQ-12^f^ scores, mean (SD)	1.9 (2.8)	1.9 (2.5)	1.2 (2.2)	1.2 (2.0)	1.4 (2.4)	1.7 (2.8)	1.0 (2.2)	0.9 (1.9)
Baseline CES-D^g^ scores, mean (SD)	—	—	—	—	2.1 (2.3)	2.1 (2.1)	1.4 (1.8)	1.3 (1.7)

Data in the table are percentages for categorical variables and means (SD) for continuous variables.

^a^The threshold between moderate and heavy drinking is ≥20 g/day in men and ≥10 g/day in women.

^b^MEDAS: Mediterranean Diet Adherence Screener (range 0–14).

^c^BMI: Body Mass Index.

^d^Ischemic heart disease, stroke or heart failure.

^e^GDS: Geriatric Depression Scale-10 item version.

^f^GHQ-12: General Health Questionnaire-12 item version.

^g^CES-D Center for Epidemiologic Studies Depression Scale-8 item.

In the Seniors-ENRICA study, drinkers with a preference for wine, who drank only with meals, or who presented a MDP pattern were more likely to be female, slightly older, more physically active, and less frequently obese than their counterparts (Table 2). Those same three types of drinker had a lower prevalence of smoking, higher MEDAS scores, a lower prevalence of respiratory disease, and a higher prevalence of cardiovascular disease, diabetes and osteomuscular disease than their counterparts. The most favorable scores in both the GDS-10 and the GHQ-12 were observed among drinkers with a preference for alcoholic beverages other than wine, those who drank with and outside of meals, and those with no MDP (Table 2).

**Table 2:** Characteristics of alcohol drinkers by alcohol consumption patterns in the Seniors-ENRICA cohort.

	Seniors-ENRICA						
	Beverage preference		Drinking with meals			MDP	
	Other	Wine	With meals	With and outside meals	Without meals	No MDP	MDP
Female, %	32.5	42.6	47.6	22.5	45.2	32.4	49.5
Age, years, mean (SD)	70.4 (5.5)	71.0 (5.9)	70.1 (5.8)	70.4 (5.8)	70.6 (5.3)	70.5(5.6)	71.3 (6.0)
Educational level, %							
≤Primary	43.2	44.7	47.0	41.5	40.6	41.9	47.8
Secondary	27.9	28.7	27.8	27.0	31.0	27.4	29.1
University	29.9	26.6	25.2	31.5	28.4	30.7	23.1
Current smokers, %	13.3	11.3	9.3	14.2	16.1	13.9	9.0
Physical activity, METs^a^ *h/wk, mean (SD)	51.5 (26.8)	56.1 (30.6)	56.5 (30.4)	52.5 (28.9)	52.1 (26.8)	52.1 (28.1)	58.2 (30.9)
TV viewing, h/wk, mean (SD)	19.2 (10.1)	18.6 (10.1)	19.5 (10.3)	18.2 (9.7)	17.9 (10.2)	18.6 (9.8)	19.3 (10.6)
MEDAS^b^ score, mean (SD)	3.9 (1.5)	4.0 (1.6)	4.1 (1.6)	3.9 (1.5)	3.9 (1.6)	3.9 (1.5)	4.1 (1.6)
Energy intake, kcal/d, mean (SD)	2172 (646)	2085 (542)	2034 (429)	2244 (658)	2115 (768)	2183 (653)	2010 (430)
BMI^c^ ≥30 kg/m^2^, %	33.7	29.2	30.7	30.9	31.0	32.6	28.0
Comorbities, %							
Cardiovascular disease^d^	5.0	5.1	5.4	5.1	3.9	4.6	5.7
Respiratory disease	8.6	7.3	7.2	7.4	10.3	8.6	6.5
Osteomuscular disease	50.0	56.1	59.4	45.3	54.2	48.9	62.0
Diabetes	14.5	16.3	16.7	14.8	14.2	14.2	17.9
Cancer	1.8	2.0	2.1	1.6	1.9	1.7	2.2
Sleeping pills, %	16.6	19.4	19.2	14.8	23.2	17.2	20.4
Other central nervous system-acting drugs,%	24.9	27.2	27.8	21.2	32.3	25.0	28.5
Baseline GDS-10^e^ scores, mean (SD)	0.6 (1.3)	0.8 (1.4)	0.8 (1.4)	0.6 (1.1)	0.8 (1.6)	0.7 (1.3)	0.8 (1.4)
Baseline GHQ-12^f^ scores, mean (SD)	1.1 (2.2)	1.3 (2.2)	1.3 (2.2)	0.9 (1.9)	1.6 (2.6)	1.2 (2.2)	1.3 (2.2)

Data in the table are percentages for categorical variables and means and standard deviations (SD) for continuous variables.

^a^MDP Mediterranean drinking pattern.

^b^METs: Metabolic Equivalents of Task.

^c^MEDAS: Mediterranean Diet Adherence Screener (range 0–14).

^d^BMI: Body Mass Index.

^e^Ischemic heart disease, stroke or heart failure.

^f^GDS: Geriatric Depression Scale-10 item version.

^g^GHQ-12: General Health Questionnaire-12 item version.

The distributions of the GDS-10, CES-D, and GHQ-12 scores at baseline and at follow-up can be observed in Fig. 1. As aforementioned, the most common score in all scales was zero and distributions were all highly skewed to the right. After a 2.8 (ENRICA) and a 7.4-year-mean follow-up (ELSA), 4% and 10% of participants developed incident depression (GDS or CESD scores >3), respectively. Table 3 displays the results for the prospective association between alcohol consumption patterns and follow-up GDS-10 and GHQ-12 scores in the Seniors-ENRICA study. Compared to never-drinkers, moderate alcohol consumers showed similar scores in the GDS-10 (PRR: 1.03; 95%CI: 0.84–1.26) and GHQ-12 (PRR: 0.88; 95%CI: 0.73–1.06). Alcohol consumption patterns, including the MDP, were not associated with GDS-10 or GHQ-12 scores. However, it is worth noting that drinkers with a wine preference showed a non-statistically significant increased number of psychological distress symptoms (PRR: 1.31; 95%CI: 1.03–1.66).

**Table 3:** Prospective association between alcohol consumption patterns and Geriatric Depression Scale (GDS) and the General Health Questionnaire-12 (GHQ-12) scores for older adults followed-up for 2.8 years; Seniors-ENRICA cohort study.

GDS					GHQ-12				
	n	%	Model 1 PRR (95% CI)	Model 2 PRR (95% CI)		n	%	Model 1 PRR (95% CI)	Model 2 PRR (95% CI)
**Average alcohol consumption**	**1200**				**Average alcohol consumption**	**1428**			
Never drinker	315	26.2	Ref.	Ref.	Never drinker (n = 383)	383	26.8	Ref.	Ref.
Ex-drinker	78	6.5	0.88 (0.57–1.34)	0.97 (0.69–1.37)	Ex-drinker (n = 94)	94	6.6	1.13 (0.82–1.57)	1.11 (0.82–1.51)
Moderate drinker	746	62.2	0.99 (0.77–1.28)	1.03 (0.84–1.26)	Moderate drinker (n = 880)	880	61.6	0.91 (0.75–1.12)	0.88 (0.73–1.06)
Heavy drinker	61	5.1	0.65 (0.27–1.53)	0.88 (0.56–1.36)	Heavy drinker (n = 71)	71	5.0	0.68 (0.42–1.09)	0.70 (0.45–1.08)
**Beverage preference**	**807**				**Beverage preference**	**951**			
Other	285	35.3	Ref.	Ref.	Other	338	33.5	Ref.	Ref.
Wine	522	64.7	0.97 (0.74–1.29)	1.17 (0.91–1.50)	Wine	613	64.5	1.31 (0.98–1.74)	1.31 (1.03–1.66)
**Drinking with meals**	**807**				**Drinking with meals**	**951**			
Only with meals	409	50.7	Ref.	Ref.	Only with meals	485	51.0	Ref.	Ref.
With and outside of meals	261	32.3	0.81 (0.53–1.25)	0.91 (0.67–1.22)	With and outside of meals	311	32.7	0.96 (0.70–1.31)	0.90 (0.67–1.19)
Only without meals	137	17.0	0.87 (0.52–1.45)	0.84 (0.61–1.18)	Only without meals	155	16.3	1.09 (0.76–1.56)	0.91 (0.66–1.25)
**Mediterranean drinking pattern (MDP)** ^**a**^	**1200**				**Mediterranean drinking pattern (MDP)** ^**a**^	**1428**			
Never drinker	315	26.2	Ref.	Ref.	Never drinker	383	26.8	Ref.	Ref.
Ex-drinker	78	6.5	0.90 (0.60–1.36)	0.96 (0.68–1.35)	Ex-drinker	94	6.6	1.13 (0.81–1.57)	1.10 (0.81–1.50)
Drinker with no MDP	746	62.2	0.92 (0.67–1.26)	0.97 (0.75–1.26)	Drinker with no MDP	583	40.8	0.89 (0.68–1.15)	0.88 (0.70–1.13)
Drinker with MDP	61	5.1	1.08 (0.81–1.43)	1.15 (0.91–1.46)	Drinker with MDP	368	25.8	1.02 (0.80–1.31)	0.98 (0.78–1.23)

PRR: Prevalence rate ratio; CI: Confidence interval.

^a^Moderate alcohol consumption with preference for wine and drinking only with meals.

Model 1: Adjusted for the age, sex, educational level (≤primary, secondary, university) and baseline: Geriatric Depression Scale or General Health Questionnaire −12 scores.

Model 2: Additionally adjusted for: tobacco smoking (never/former/current), physical activity (MET-h/week), time watching TV (h/week), MEDAS index (excluding alcohol), total energy intake (Kcal/day), Body Mass Index (≤25, 25–29.9, ≥30 kg/m^2^), cardiovascular disease, respiratory disease, osteo-muscular disease, diabetes, cancer, sleeping pills and central nervous system-acting medications.

Models for specific drinking habits (i.e. beverage preference, drinking with meals or the MDP) were further adjusted for average alcohol consumption (grams/day).

In the Seniors-ENRICA, we found no evidence of an association between moderate alcohol intake (IRR: 1.14; 95%CI: 0.57–2.29) or the MDP (IRR: 1.84; 95%CI: 0.80–4.25) and incidence of self-reported depression (defined as a physician-diagnosis of depression or as being on antidepressant medication) (Supplementary Table 3). Results for heavy drinking, beverage preference, or drinking with meals and self-reported depression could not be interpreted due to a low number of incident self-reported depression cases (Supplementary Table 3).

Results for the prospective association between average alcohol consumption and follow-up CES-D and GHQ-12 scores in the ELSA cohort can be found in Table 4. Compared to never drinkers, both moderate and heavy drinkers showed similar percent changes in depression and psychologic wellbeing scales. In this sense, the PRR (95%CI) for CESD-D was 0.92 (0.79–1.06) in moderate drinkers, and 1.00 (0.84–1.20) in heavy drinkers; the corresponding results for the GHQ-12 were 0.75 (0.55–1.01) and 0.84 (0.58–1.21), respectively.

**Table 4:** Prospective association between average alcohol consumption and Center for Epidemiologic Studies Depression Scale (CES-D) and the General Health Questionnaire-12 (GHQ-12) scores for older adults followed-up for 7.4 years; English Longitudinal Study of Ageing (ELSA).

CESD					GHQ-12				
	n	%	Model 1 PRR (95% CI)	Model 2 PRR (95% CI)		n	%	Model 1 PRR (95% CI)	Model 2 PRR (95% CI)
**Average alcohol consumption**	**3971**				**Average alcohol consumption**	**3360**			
Never drinker	219	5.5	Ref.	Ref.	Never drinker	161	4.8	Ref.	Ref.
Ex-drinker	188	4.7	1.16 (0.96–1.41)	1.11 (0.91–1.35)	Ex-drinker	156	4.6	0.87 (0.59–1.30)	0.81 (0.54–1.21)
Moderate drinker	3146	79.2	0.95 (0.83–1.09)	0.92 (0.79–1.06)	Moderate drinker	2688	80.0	0.77 (0.57–1.04)	0.75 (0.55–1.01)
Heavy drinker	418	10.5	1.05 (0.88–1.26)	1.00 (0.84–1.20)	Heavy drinker	355	10.6	0.88 (0.61–1.27)	0.84 (0.58–1.21)

PRR: Prevalence rate ratios; CI: Confidence interval.

Model 1: Adjusted for the age, sex, educational level (≤primary, secondary, university) and baseline CESD scores or GHQ-12 scores.

Model 2: Adjusted additionally for tobacco smoking (never/former/current), physical activity, BMI (≤25, 25–29.9, ≥30 kg/m^2^), cardiovascular disease, respiratory disease, osteomuscular disease, diabetes, and cancer.

Sensitivity analyses yielded similar results to those presented in the main tables (data not shown).

## Discussion

Overall, we found no consistent protective association between moderate alcohol consumption and depression in older adults. Moreover, in the Seniors-ENRICA cohort, adherence to certain alcohol consumption patterns such as drinking only with meals or the MDP failed to show an association with either of the studied mental health outcomes. However, results showed some non-significant tendency to a lower risk of depression (Spanish sample) and psychological distress (both samples) associated with heavy drinking, which should be further investigated in studies with a larger number of heavy drinkers.

Previous research on the link between moderate alcohol intake and mental health in adults has yielded inconsistent results. In the National Psychiatric Morbidity Survey in England, which followed 2,413 adults for 18 months, moderate drinking (defined as drinking <21 or <14 units/week in men or women, respectively, or having an AUDIT score <8 points), showed no association with incident anxiety or depression (measured with the Clinical Interview Schedule-Revised instrument) when compared to abstaining^17^. By contrast, Norway’s Nord-Trøndelag Health Survey of 38,930 individuals with a mean age of 48 years, ascertained that low-to-moderate drinkers had a lower risk of case-level anxiety and depression (based on Hospital Anxiety and Depression Rating Scale) than non-drinkers (self-reported abstainers and non-abstainers currently consuming no alcohol). Unfortunately, the study’s partial cross-sectional design hindered drawing causal inference^39^. Also, a Spanish cohort study of 13,619 university graduates (mean age: 38 years) followed for 10 years showed that female – but not male- moderate drinkers (5–15 g/day) were less likely to self-report suffering from depression than abstainers^15^. However, a study of midlife alcohol consumption of the Whitehall II cohort of British civil servants, concluded that there was no association between drinking patterns, including a moderate (<40 g/day) one, and risk of depression. The 28-year follow-up study consisted of 7,478 men and women between the ages of 35 and 55 years and depression was assessed by the GHQ-30^40^. More recently, results based on 3,201 individuals aged 18–65 years, who were primary care attenders in the WHO collaborative study of Psychological Problems in General Health Care, suggested a possible protective effect of moderate (defined as AUDIT <8 points) drinking in the risk of developing a new onset of depression and generalized anxiety disorder (GAD)^14^.

To our knowledge only one study has focused on older adults. Gea *et al*. followed 5,505 high-risk men and women (55 to 80 y) from the PREDIMED Trial. Unlike the WHO collaborative study, participants in PREDIMED were initially free of depression or a history of depression, and did not have any history of alcohol-related problems. After 7 years of follow-up, moderate drinkers (5–15 g/day) of both genders were less likely to self-report a diagnosis of depression than abstainers^16^. Specifically, wine consumption in the range of 2–7 drinks/week decreased the risk of incident depression in this population^16^. Results in our study using abstainers (never drinkers and ex-drinkers) as the reference category or changing the threshold between moderate and heavy intake to 15 g/day showed no association between moderate alcohol intake and depression. A partial explanation for the discrepant results between the PREDIMED study and ours might be that participants in the PREDIMED trial were primary-care patients participating in an intensive group-based health education intervention; thus, we speculate that the intervention-related social support very likely geared towards a greater adherence to a Mediterranean diet (including moderate drinking)^41^, received by PREDIMED participants may have contributed to a lower depression risk^42^. Moreover, greater adherence to a healthy diet also reduces the incidence of cardiovascular disease, diabetes and other chronic diseases which, in turn, are strong risk factors or triggers for depression.

In some cases, differences between clinic-based studies^14,16^ -which found a protective association between moderate alcohol intake and mental health- and studies in community samples^40^ which did not find such association (i.e. ours), could be partially explained by the self-medication hypothesis, whereby alcohol intake is used to ameliorate mental and other health problems; this phenomenon could operate more strongly in clinical than community samples due to their worse health status.

Our work has several strengths compared to published research. First, unlike previous studies^14–16^, we distinguished between never drinkers and ex-drinkers. Since ex-drinkers might have quit drinking due to ill health, including depression, studies incorporating ex-drinkers in the reference category may overestimate the protective effect of moderate alcohol drinking on depression. Second, we adjusted our analyses for a larger number of lifestyle and morbidity variables than previous reports^14,15,17^. Third, as far as we are aware of, this is the first study to provide detailed analyses on the association between different patterns of moderate alcohol consumption, including the MDP, and mental health outcomes^16^. Fourth, we used a validated diet history to estimate alcohol consumption in the Seniors-ENRICA cohort. In fact, the Pearson correlation coefficient between alcohol consumption data from the diet history and seven 24-h recalls over the course of one year was a satisfactory 0.65^32^. Finally, we obtained consistent results using two cohorts characterized by different patterns of alcohol consumption and different follow-up periods. This is important because it suggests that the association, or lack thereof, between moderate alcohol intake and depression does not differ by cultural and social context, i.e., it may be similar in “wet and “dry” countries. Additionally, the ELSA 7.4-year mean follow-up suggests that the failure to detect an association is not due to failing to follow individuals “long enough to see an effect”.

The main limitation of the study was that alcohol consumption was self-reported with the corresponding risk of recall error and social desirability bias. It is known that questions on alcohol habits are commonly subject to under-reporting, particularly among heavy drinkers^43^. In this sense, although numbers were similar to those reported in previous population-based studies^44,45^, the percentage of heavy drinkers in our cohorts was rather low (5% in Spain and 10% in the UK). In any case, there is evidence that telephone interviews are less affected by social desirability bias than personal interviews. Second, we lacked repeated measurements of alcohol intake during follow-up to update the information on exposure to alcohol. Third, although we used validated depression and psychological distress scales, these were not confirmed by a clinical diagnosis. Thus, we cannot rule out the existence of diagnostic bias, which effect on these results remains unknown. However, in the Seniors-ENRICA, were we had information on previous physician-diagnoses of depression or being under antidepressant treatment (which was confirmed against drug packages at home), we observed a similar prevalence of depression either using this information or the GDS scale. Fourth, our results may not be generalizable to institutionalized older adults. Finally, in both cohorts we observed an association between heavy drinking and decreased number of psychological distress symptoms approaching statistical significance. However, the small number of heavy drinkers in this cohort makes these results unreliable.

## Conclusions

Our results do not support the presence of a protective effect derived from moderate alcohol consumption in general, and wine in particular, on the risk of developing depression or psychological distress among older adults. Given the inconsistency in this literature on the general population and the scarce research on older adults, future research should assess whether and how changes in alcohol consumption patterns affect depression risk using repeated measures of alcohol intake and clinical diagnoses of depression in this vulnerable population.

## Electronic supplementary material

Supplementary Information - Prospective association between alcohol consumption patterns and the risk of self-reported incident depression in older adults
