# Supplementary material for: Moderate alcohol drinking is not associated with risk of depression in older adults
Source: Sci Rep. 2018 Jul 31;8:11512. doi: 10.1038/s41598-018-29985-4 (PMC6068095; doi:10.1038/s41598-018-29985-4)
Supplement: Supplementary file 1 — Supplementary Information - Prospective association between alcohol consumption patterns and the risk of self-reported incident depression in older adults [file 41598_2018_29985_MOESM1_ESM.docx]

**Moderate alcohol drinking is not associated with risk of depression in older adults.** Esther García-Esquinas, Rosario Ortolá, Iñaki Galán, Hosanna Soler-Vila, Martín Laclaustra, Fernando Rodríguez-Artalejo.

**Supplementary table 1. Prospective association between alcohol consumption patterns and the risk of self-reported incident depression in older adults**

|  |  |  |  |  |
| --- | --- | --- | --- | --- |
|  | **n/Total** | **Model 1 Incidence rate ratio**  **(95% CI)** | **Model 2 Incidence rate ratio**  **(95% CI)** |  |
| **Average alcohol consumption** | **45/1112** |  |  |  |
| Never drinker | 16/282 | Ref. | Ref. |  |
| Ex-drinker | 4/66 | 1.44 (0.52-3.98) | 1.32 (0.41-4.27) |  |
| Moderate drinker | 24/707 | 1.14 (0.57-2.29) | 1.13 (0.55-2.31) |  |
| Heavy drinker | 1/57 | 0.74 (0.10-5.46) | 0.77 (0.10-5.98) |  |
| **Beverage preference** | **25/764** |  |  |  |
| Other | 6/273 | Ref. | Ref. |  |
| Wine | 19/491 | 1.79 (0.68-4.72) | 1.56 (0.55-4.38) |  |
| **Drinking with meals** | **25/764** |  |  |  |
| Only with meals | 13/384 | Ref. | Ref. |  |
| With and outside of meals | 6/252 | 1.52 (0.62-3.75) | 1.41 (0.49-4.05) |  |
| Only without meals | 6/128 | 0.76 (0.29-2.03) | 0.84 (0.28-2.54) |  |
| **Mediterranean drinking pattern (MDP)^a^** | **45/1112** |  |  |  |
| Never drinker | 16/ 282 | Ref. | Ref. |  |
| Ex-drinker | 4/66 | 1.29 (0.44-3.72) | 1.24 (0.38-4.06) |  |
| Drinker with no MDP | 13/476 | 1.51 (0.68-3.33) | 0.47 (0.65-3.34) |  |
| Drinker with MDP | 12/288 | 1.86 (0.81-4.27) | 1.84 (0.80-4.25) |  |

CI: Confidence interval.

^a^ Moderate alcohol consumption with preference for wine and drinking only with meals.

Model 1: Adjusted for the age, sex and educational level (≤primary, secondary, university).

Model 2: Additionally adjusted for: tobacco smoking (never/former/current), physical activity (MET-h/week), time watching TV (h/week), MEDAS index (excluding alcohol), total alcohol consumption (grams/day), total energy intake (Kcal/day), Body Mass Index (≤25, 25-29.9, ≥30 kg/m^2^), cardiovascular disease, respiratory disease, osteo-muscular disease, diabetes, cancer, sleeping pills and central nervous system-acting medications.

**Supplementary table 2. Association between alcohol consumption patterns and changes in the Geriatric Depression Scale (GDS) and the General Health Questionnaire-12 (GHQ-12) scores in older adults followed-up during 2.8 years; Seniors-ENRICA cohort study**

|  | **GDS** | |  | **GHQ-12** | | |
| --- | --- | --- | --- | --- | --- | --- |
|  | **Model 1 ß (95% CI)^a^** | **Model 2  ß (95% CI)** |  |  | **Model 1 ß (95% CI)** | **Model 2  ß (95% CI)** |
| **Average alcohol consumption (n=1200)** |  |  |  | **Average alcohol consumption (n=1428)** |  |  |
| Never drinker (n=315) | Ref. | Ref. |  | Never drinker (n=383) | Ref. | Ref. |
| Ex-drinker (n=78) | -0.21 (-0.81; 0.39) | -0.15 (-0.74; 0.44) |  | Ex-drinker (n=94) | 0.14 (-0.47; 0.74) | 0.22 (-0.39; 0.82) |
| Moderate drinker (n=746) | 0.16 (-0.08;0.40) | 0.16 (-0.07;0.40) |  | Moderate drinker (n=880) | -0.10 (-0.45;0.26) | -0.09 (-0.45; 0.27) |
| Heavy drinker (n=61) | 0.28 (-0.00;0.58) | 0.25 (-0.06; 0.56) |  | Heavy drinker (n=71) | -0.27 (-0.97; 0.43) | -0.31 (-1.01; 0.38) |
| **Beverage preference (n=807)** |  |  |  | **Beverage preference (n=951)** |  |  |
| Other (n=285) | Ref. | Ref. |  | Other (n=338) | Ref. | Ref. |
| Wine (n=522) | 0.01 (-0.16; 0.17) | -0.01 (-0.16;0.14) |  | Wine (n=613) | 0.19 (-0.10; 0.49) | 0.21 (-0.09; 0.51) |
| **Drinking with meals (n=807)** |  |  |  | **Drinking with meals (n=951)** |  |  |
| Only with meals (n=409) | Ref. | Ref. |  | Only with meals (n=485) | Ref. | Ref. |
| With and outside of meals (n=261) | -0.05 (-0.23;0.12) | -0.11 (-0.29; 0.07) |  | With and outside of meals (n=311) | 0.04 (-0.30;0.39) | 0.01 (-0.35; 0.37) |
| Only without meals (n=137) | -0.15 (-0.45;0.16) | -0.08 (-0.36; 0.21) |  | Only without meals (n=155) | -0.22 (-0.63;0.20) | -0.16 (-0.57;0.24) |
| **Mediterranean drinking pattern (MDP)^b^ (n=1200)** |  |  |  | **Mediterranean drinking pattern (MDP)^a^ (n=1428)** |  |  |
| Never drinker (n=315) | Ref. | Ref. |  | Never drinker (n=383) | Ref. | Ref. |
| Ex-drinker (n=78) | -0.21 (-0.81; 0.38) | -0.15 (-0.73; 0.44) |  | Ex-drinker (n=94) | 0.12 (-0.48; 0.73) | 0.20 (-0.40;0.81) |
| Drinker with no MDP (n= 500) | 0.13 (-0.11; 0.38) | 0.11 (-0.16; 0.37) |  | Drinker with no MDP (n= 583) | -0.13 (-0.57;0.31) | -0.12 (-0.55; 0.30) |
| Drinker with MDP (n=307) | 0.22 (-0.05; 0.48) | 0.19 (-0.07; 0.46) |  | Drinker with MDP (n=368) | 0.01 (-0.41;0.44) | 0.04 (-0.39; 0.46) |

^a^ Confidence intervals were calculated using bootstrap methods.

^b^ Moderate alcohol consumption with preference for wine and drinking only with meals.

Model 1: Adjusted for the age, sex, educational level (≤primary, secondary, university)

Model 2: Additionally adjusted for: tobacco smoking (never/former/current), physical activity (MET-h/week), time watching TV (h/week), MEDAS index (excluding alcohol), total alcohol consumption (grams/day), total energy intake (Kcal/day), Body Mass Index (≤25, 25-29.9, ≥30 kg/m^2^), cardiovascular disease, respiratory disease, osteo-muscular disease, diabetes, cancer, sleeping pills and central nervous system-acting medications.

**Supplementary table 3. Association between average alcohol consumption and changes in the Center for Epidemiologic Studies Depression Scale (CES-D) and the General Health Questionnaire-12 (GHQ-12) scores in older adults followed-up for 7.4 years; English Longitudinal Study of Ageing (ELSA) cohort.**

|  | **CESD** | |  | | **GHQ-12** | | |
| --- | --- | --- | --- | --- | --- | --- | --- |
|  | **Model 1 ß (95% CI)** ^a^ | **Model 2  ß (95% CI)** |  |  | | **Model 1 ß (95% CI)** | **Model 2  ß (95% CI)** |
| **Average alcohol consumption (n=3971)** |  |  |  | **Average alcohol consumption (n=3360)** | |  |  |
| Never drinker (n=219) | Ref. | Ref. |  | Never drinker (n=161) | | Ref. | Ref. |
| Ex-drinker (n=188) | 0.17 (-0.27; 0.62) | 0.15 (-0.29; 0.59) |  | Ex-drinker (n=156) | | -0.43 (-1.10; 0.23) | -0.44 (-1.08; 0.20) |
| Moderate drinker (n=3146) | 0.04 (-0.24;0.31) | 0.01 (0.25; 0.28) |  | Moderate drinker (n=2688) | | -0.32 (-0.72;0.08) | -0.33 (-0.72; 0.07) |
| Heavy drinker (n=418) | 0.11 (-0.20;0.43) | 0.09 (-0.22;0.40) |  | Heavy drinker (n=355) | | -0.06 (-0.59;0.47) | -0.05 (-0.57; 0.47) |

^a^ Confidence intervals were calculated using bootstrap methods.

Model 1: Adjusted for the age, sex, educational level (≤primary, secondary, university)

Model 2: Adjusted additionally for tobacco smoking (never/former/current), physical activity, BMI (≤25, 25-29.9, ≥30 kg/m^2^), cardiovascular disease, respiratory disease, osteomuscular disease, diabetes, and cancer.
